# Uncovering hidden prosocial behaviors underlying aggression motivation in mice and young children

**DOI:** 10.1186/s12993-024-00260-z

**Published:** 2024-11-28

**Authors:** Chih-Lin Lee, Yu-Shan Su, Chi-Yu Chang, Tzu-Yun Kung, Yu-Kai Ma, Pei-Yun Zeng, Ching-Chuan Cheng, Yu-Jen Chang, Yu-Ju Chou, Tsung-Han Kuo

**Affiliations:** 1 Institute of Systems Neuroscience, National Tsing Hua University, Hsinchu, Taiwan, Republic of China; 2 Department of Early Childhood Education, National Tsing Hua University, Hsinchu, Taiwan, Republic of China; 3 Department of Educational Psychology and Counseling, National Tsing Hua University, Hsinchu, Taiwan, Republic of China; 4 Department of Life Science, National Tsing Hua University, Hsinchu, Taiwan, Republic of China; 5 Dadong Elementary School, Kaohsiung, Taiwan, Republic of China; 6 Brain Research Center, National Tsing Hua University, Hsinchu, Taiwan, Republic of China

**Keywords:** Social behaviors, Social aggression, Prosocial behaviors, Mouse allogrooming

## Abstract

**Background:**

Animals exhibit a wide range of social behaviors, including positive actions that promote social cohesion and negative behaviors associated with asserting dominance. While these behaviors are often viewed as opposites, they can also exist independently or coexist in complex ways, necessitating further investigation into their interrelationships.

**Results:**

To study the interplay between these two types of behaviors, we examined mouse social behaviors using resident-intruder assays and revealed a negative correlation between social aggression and prosocial allogrooming. Suppressing aggressive motivation through various manipulations, including social subordination, olfaction ablation, and inhibition of aggressive neural circuits, led to an increased display of allogrooming behavior. The mouse findings prompted us to further explore the relationship between aggression and prosocial behaviors in preschool children. Similarly, we observed a negative association between aggression and prosocial behaviors, which were potentially influenced by their inhibitory control abilities.

**Conclusions:**

Through this cross-species study, we uncovered the inhibitory impact of aggressive neural circuits on mouse allogrooming and established a link between aggression and prosocial behaviors in children. These insights offer valuable implications for understanding and potentially influencing social interactions in both animal and human contexts, with potential applications in preschool education practices.

**Supplementary Information:**

The online version contains supplementary material available at 10.1186/s12993-024-00260-z.

## Introduction

Humans and other animals display various social behaviors with different emotional valences or biological functions. Positive behaviors, such as prosocial actions and allogrooming, promote cooperation and bonding among individuals, while negative behaviors, like aggression and avoidance, serve as defense mechanisms or assert dominance in social conflicts [1, 2]. The relationships between these two types of behaviors are intricate and multifaceted. While it is generally believed that positive and negative behaviors are opposites [1, 3], they are not always negatively associated and can sometimes be independent or even coexist [4–6], depending on various factors like motivations or cultural norms.

In humans, the association between prosocial behavior and aggression changes over time [7, 8]. While studies with infants show no correlations, they became negatively correlated after the age of 3 [8]. The negative correlations between aggression and prosocial behaviors have also been reported in some studies of teenagers or school-age children [1, 3], but others have shown single-direction, coexisting, or complicated mutual relationships [4–6, 9, 10]. Considering that subjects in these studies have gone through school education and peer interactions for many years, it is more complicated and challenging to clarify the association between the two [11]. Therefore, focusing on preschool young children, who have a relatively stable physical and mental state but with limited exposure to school education and peers, may provide more insights into the intricacies of their relationship.

Social behaviors have also been studied extensively in house mice, *Mus musculus*, a widely used model organism in laboratories. Under the resident-intruder assay [12], resident male mice singly housed for at least one week display aggressive attacks toward male intruders [13]. The underlying molecular mechanisms and neural circuits involved in aggression have been explored in both basic and translational studies [14, 15]. Several manipulations have been devised to induce or inhibit aggressive behaviors in mice. For instances, it has been reported that the male aggression can be influenced by social status [16]. Ablation of olfactory systems, mainly MOE (main olfactory epithelium) and VNO (vomeronasal organ), eliminates male aggression [15]. Multiple brain regions, including MeApd (posterodorsal part of the medial amygdala) and VMHvl (ventrolateral part of the ventromedial hypothalamus), are also known to regulate mouse aggression [15]. However, it is important to note that aggression is not the sole social behavior during same-sex interaction. Other behaviors, like allogrooming or social investigation, may possess completely distinct emotional valence or biological significance.

Allogrooming is a significant social behavior observed across many animal species, playing a key role in maintaining and strengthening social bonds [17]. Groomers invest time and effort in grooming others, making this behavior widely recognized as cooperative or prosocial in many species of primates and rodents [2, 18–25], including house mice [26, 27]. Although allogrooming in mice has been documented for decades [28], it has received limited attention within the behavioral neuroscience community, particularly in studies using the resident-intruder assay. This may be attributed to the predominant focus on aggressive behaviors during these interactions, which often overshadows the observation and analysis of other social behaviors. By shifting attention to non-aggressive mice during social encounters, we may gain a more comprehensive understanding of the full spectrum of social interactions, including the role of allogrooming and its relationship to other behaviors in these dynamics.

Since aggression and prosocial behaviors in mice have not been explored in parallel, their relationship remains largely unexamined, leaving open questions about how these two behaviors influence one another. In this study, using various approaches, we explored how suppressing aggression affects allogrooming as well as the correlation between these two types of behaviors in male mice. To test the generalizability of our findings on mice, we also conducted a study on the relationship between aggression and prosocial behaviors in preschool children. Our study may provide valuable insights into the intricacies of social interactions in both species and could have important implications for preschool education and understanding early social development.

## Materials and methods

### Mice

C57BL/6J and BALB/cByJ male mice were obtained from the National Laboratory Animal Center in Taiwan. C57BL/6N mice were purchased from BioLASCO Taiwan company, Ltd. With the exception of C57BL/6N mice, which were used for stereotactic surgery, C57BL/6J was used as residents for all experiments. Mice used in social hierarchy experiments and distressed social partners were housed in pairs, whereas mice in all other experiments were individually housed. While all C57BL/6 residents were used for only one experiment, BALB/cByJ mice, housed in groups of four, were repeatedly used as intruders from 8 weeks old until they reached one year of age. All animal procedures followed institutional guidelines established and approved by the Institutional Animal Care and Use Committee of National Tsing Hua University. The strains, mouse numbers and manipulations for each experiment were listed in Table S1.

### Ablation of the main olfactory epithelium (MOE)

2,6-dichlorobenzonitrile (dichlobenil) has been widely used to damage olfactory epithelium [29, 30]. Mice received intraperitoneal injections of dichlobenil (Sigma, D57558) at a concentration of 50 mg/mL, dissolved in dimethyl sulfoxide (DMSO) (Sigma-Aldrich, V900090), at a dosage of 100 µg/g body weight. The injections were administrated on days 1, 3 and 5 prior to the experimentation [31]. Control animals were injected with DMSO only.

### Mouse behavioral assays

#### Establishment and identification of social rank (Fig. S1)

Eight-week-old male mice were housed in pairs for 1 week to form a social hierarchy (Hierarchy Establishment). We modified the standard resident-intruder assay to identify the social ranks of two resident mice (Hierarchy Identification). A BALB/cByJ intruder mouse with ablated MOE was introduced into the cage for a 10-minute interaction. The resident that carried out more than one attack on the intruder was identified as the dominant male, whereas the resident that showed no aggression was identified as the subordinate. Pairs of mice that both showed aggression were not used. Pairs of mice in which neither showed aggression were tested again the following day; if both males continued not showing aggression, they were also excluded. Identified dominant and subordinate males were used separately for the standard resident-intruder assay (Behavioral Testing).

#### Tube test behavioral assay

The tube test was performed based on a previous study [32]. Mice were habituated to the procedure room for 1 h on two consecutive days and were tested on the third day. A tube test trial was carried out involving two mice that were simultaneously released at opposite ends of a clear Plexiglas tube (3.75 cm diameter, 60 cm length) and then ran toward the middle. When a mouse retreated and placed all four paws outside the tube, the trial was considered over, and that mouse was classified as the loser. Four consecutive trials were conducted for each pair. A mouse was defined as dominant if it won 3 (75%) or 4 (100%) out of 4 trials, while a subordinate mouse was defined by winning 0 or 25% of the trials. Pairs without a winner (50%) were removed. The interior of the tube was cleaned with 70% ethanol after each pairwise trial.

#### Standard resident-intruder assay

The resident-intruder assay was carried out as previously described [12]. In brief, with the exception of pair-housed males for social hierarchy or distressed social partners, all resident males were individually housed in standard rack cages for a minimum of 7 days. Prior to the assay, mice were moved to the procedure room for a period of 1-hour on two consecutive days to acclimate the environment. On the third day, following a 1-hour habituation period in procedure room, an intruder was introduced into the home cage of a resident mouse. Their interaction was digitally recorded for 10 min. Both the habituation and the assay were conducted under red light conditions to minimize visual cues. All intruders used in experiments were adult BALB/cByJ treated with dichlobenil to ablate the MOE and eliminate aggression.

The recorded videos were subsequently analyzed manually with the assistance of software BORIS to obtain the total time of aggression (tail rattling, attack biting, wrestling and chasing) [33], allogrooming (mouthing, licking, nibbling but not barbering) and social investigation (encompassing all social interactions excluding aggression, allogrooming and mounting) exhibited by each resident. Parameters such as latency (time until the first occurrence) and number of bouts (instances of continuous action) for each behavior were also calculated. For mice that did not engage in a specific behavior within the 10-minute assay period, the latency was denoted as 600 s. In addition, to capture the recipients’ responses during interaction, we recorded their stationary behaviors (defined as no movement for more than 3 s) and categorized them as either freezing-like (upright posture, hunched back, or motionless immediately after fleeing) or non-freezing-like behaviors [34–36].

#### Assays with intruders covered with foreign materials

Mineral oil (100 µl) or glue from a glue stick was spread onto one side of an intruder with MOE ablation before its use in a resident-intruder assay, which was carried out as described above.

#### Assays for evaluation of cleaning efficiency

Red poster paint (Pentel, Scarlet Lake No. 37) was carefully applied to a designated area on one side of an intruder approximately 2.4 × 1.1 cm^2^ in size. The intruder was anesthetized by intraperitoneal injection of 20 mg/kg Zoletil 50 (Virbac) to prevent self-grooming which could interfere with the paint marking. Following the application of the paint, a drying period of 10 min was allowed. Subsequently, the intruder, with dry paint, was introduced into a cage either with or without residents for a duration of 10 min. After the completion of the assay, a photograph was taken, and the intensity of the red poster paint color was measured using ImageJ software.

#### Two-choice assay

Two stimuli, mineral oil (100 µl, Sigma, M5904) or glue from a glue stick (Simbalion, GS104), and 100 µl 1× PBS (as a control) were individually applied to 1 × 1−inch squares of odorless blotting paper (Fisher Scientific 05-714-4). The papers were affixed to opposite walls of an arena approximately 7.5 cm from the bedding, using double-sided adhesive tape. Following a 1-hour habituation period in the procedure room, a mouse was introduced into the arena and given 10 min to explore the papers. The behaviors of mice during exploration were digitally recorded under red light conditions. The recording was subsequently used to score the investigation time of each stimulus. Investigation time was defined as the time during which the nose of the mouse was within 0.5 cm of the blotting paper.

#### Distressed social partners

The mice were housed in pairs for a minimum of 1 week prior to the experiment. Two days before the assay, the mice underwent a 1-hour habituation period each day in the procedure room. On the day of the test, the mice would once again be moved into the procedure room for 1-hour habituation period prior to the experimental trials. For distressed partners, 30 min prior to the experimental trials, mice would be taken out of their home cage and put into a 50mL centrifuge tube with breath holes and positioned stilly outside their home cage [26]. After the 30-minute restraint period, the mice would be returned to their home cage for behavioral observation. In the control (Unstressed) group, 30 min before the experiment trial commenced, the mice would be simply taken out of their home cage and placed into a neutral clean cage before being transferred back to their home cage for the behavior trials. Following the return of the mice to their home cage, the interaction between the testing mice (without manipulation) and their distressed/unstressed partners would be recorded for 10 min and subsequently analyzed manually with the assistance of software BORIS [33].

### Excitotoxicity lesion

To induce a neural lesion, ibotenic acid (IBO) at a concentration of 10 mg/mL in 10xPBS was bilaterally injected into either the MeApd (ML ± 2.10, AP-1.50, DV-5.40) or VMHvl (ML ± 0.70, AP-1.60, DV-5.55) in a volume of 200nL [37]. For the control group, 200nL of 10x PBS was injected at the same target site. Following the injection, the mice were given a minimum of 7 days to recover before behavioral testing. The efficacy of the lesion was confirmed through immunohistochemical (IHC) staining.

### Stereotaxic surgical procedures

Because the stereotaxic surgery (injection of PBS only) itself had a significant impact on C57BL/6J mouse behaviors, including reducing aggression and increasing allogrooming behaviors (Fig. S2A-D), in order to compare the behaviors of mice with and without lesion, we had to switch to C57BL/6N mice, whose behaviors were not affected significantly by the surgery (Fig. S2E-H). At 8 weeks of age, mice were anesthetized with isoflurane (5% induction, 1–1.5% maintenance) and placed in a stereotaxic platform. Ophthalmic ointment (Puralube) was applied at the beginning to protect the eyes. The head skin was sterilized with 75% ethanol, followed by careful removal using scissors. Two 1 mm micro-injection holes were drilled into the skull, and the micro-injection needle was inserted into the desired location. Once the injection was completed, the needle was withdrawn, and the holes were sealed with bone wax. To ensure stability, the head was sealed with Tempron (GC company, Japan). At the end of the procedure, carprofen (50 mg/mL, RIMADYL^®^) at a dose of 4.6 mg/kg and ampicillin at dose of 20 mg/kg by body weight were administrated subcutaneously to alleviate discomfort and prevent infection. Following the surgery, the mice were individually housed and carefully monitored to ensure proper recovery before further behavioral testing.

### Immunochemistry

The Brains were collected and pre-fixed with 4% paraformaldehyde in PBS for 24 h. After pre-fixation, the brains were cut into 50 μm thick sections using a vibratome. The brain slices were then blocked in a solution containing 3% BSA (Sigma-Aldrich, A3059) in 0.3% PBST, PBS plus Triton X-100 (Sigma-Aldrich, V900502), for an hour. Subsequently, the sections were incubated with the primary antibody Anti-NeuN Antibody (Sigma-Aldrich, MAB377) at a dilution of 1:500 for neuron cell staining in the blocking solution for 16 h at 4℃. After 3X wash in 0.1% PBST for 30 min at room temperature, the sections were incubated with the secondary antibody Goat Anti-Mouse IgG H&L (Alexa Fluor^®^ 594, Abcam, ab150120) at a dilution of 1:1000 in the blocking solution for 2.5 h at room temperature. After 2X wash in 0.1% PBST and 1X wash in PBS each for 10 min at room temperature, sections were incubated with Hoechst 33342, Trihydrochloride, Trihydrate (Invitrogen™, H3570) at a dilution of 1:10000 for 10 min at room temperature. Finally, the sections were washed in 3X in PBS for 10 min each, mounted on glass slides, and sealed with ProLong™ Diamond Antifade Mountant (Invitrogen™, P36970) for imaging. All images were captured by NIS-Element AR 5.21.03 with Nikon ECLIPSE Ni microscope and Cool SNAP HQ2 camera from Photometrics^®^. Regions of interest were circled manually and based on Mouse Brain Atlas as reference [38]. Cell numbers were counted using Image J software following the same protocol and criteria [39].

### Children participants

Approved by the National Tsing Hua University Research Ethics Committee (REC No.11206HT093), we sent research recruitment letters to 6 preschools in the Hsinchu area in Taiwan, soliciting newly enrolled children to participate in this study. A total of 118 parents’ informed consent was obtained, including 67 boys and 51 girls, with an average age of 52.31months (47 ~ 57 months).

#### Procedure

We collected one year of longitudinal data for this study. In the 4^th^ week after the preschools started, we asked the preschool teachers to fill in the three scales after they carefully observed the daily behaviors of these newly admitted young children. After one school year (12 months later), the class teachers were asked to fill in the same scales again according to the children’s daily behaviors.

### Children behavioral assessments

#### Aggressive behavior

The Aggressive Behavior Subscale of the Preschool Social Behavior Scale [40], revised from the Preschool Social Behavior Scale—Teacher Form [41], was used in this study. The scale includes 6 items in Overt Aggression Subscale (e.g. The child will kick or hit others) and 5 items in Relational Aggressive Behavior Subscale (e.g. The child will ask other children not to play with certain child). The applicable age of this scale is 3.5 ~ 5.5 years old. The scale was filled out by the class teachers based on their daily observation of the social interaction between the target child and their peers. The scale is a continuous 5-point Likert scale, ranging from “never like this”, “rarely like this”, “sometimes”, “usually” to “always”, giving 1 ~ 5 points. The total score was converted to the z score for statistical analysis. The higher the sum score of the items is, the more overt and relational aggression the children have.

#### Prosocial behavior

Preschool Prosocial Behavior Scale was used in this study [42]. This scale has 25 items and describes the four dimensions of children’s prosocial behaviors, including sharing (e.g. S/He will give her/his favorite items, such as stickers, picture cards, etc., to her/his friends.), helping (e.g. S/He will proactively provide assistance to children who have difficulties in work or activities.), caring (e.g. S/He comforts other children when they cry or get hurt.) and cooperation (e.g. S/He can work with other children to get things done.). Class teachers were asked to fill in the scale based on observations of children’s daily behavior. This scale adopts a 5-point Likert scale, ranging from 1 to 5 points from “never”, “rarely”, “sometimes”, “usually”, and “always”. The total score was converted to the z score for statistical analysis. The higher the total score is for each dimension, the greater the performance of prosocial social behavior.

#### Executive function

The Taiwanese Traditional-Chinese Childhood Executive Functioning Inventory was used to assess the self-inhibitory control of young children [43]. This scale has a national norm for ages from 4 to 12 years old. This scale is a five-point scale, from 1 to 5 points, divided into “completely incorrect”, “mostly incorrect”, “partially correct”, “correct”, and “completely correct”. Teachers were asked to choose appropriate behavior descriptions after observing children’s daily performance (e.g. Even if he is ordered to stop, it is still difficult for him to stop immediately during activities. For example, he always jumps a few more times or plays on the computer for a while after being told to stop.). Because this scale is presented with reverse questions, the total score was reversed and converted to the z score for the statistical analysis. The higher the total score is, the greater the ability of inhibitory self-control for the children.

### Statistical analyses

All statistics were carried out using GraphPad Prism 8.3 and SPSS 22.0 software. Shapiro-Wilk normality test was used to analyze the distribution of the data. For normally distributed data, Unpaired t-test (two-tailed) was used to compare two independent groups. Paired t-test (two-tailed) was applied to compare two dependent groups. Pearson correlation (with two-tailed significance testing) was applied to analyze correlation between poster paint intensity and allogrooming. For data not normally distributed, Mann‒Whitney test (two-tailed) was used for two independent data sets. Wilcoxon signed-rank test (two-tailed) was used for two dependent groups. Non-linear Spearman regression was used for correlation analyses. The relationship between social ranks identified through the intruder assay and the tube test was examined using Fisher’s Exact Test. Controlling inhibitory self-control to analyze the effect of aggression on prosocial behavior was done by hierarchical regression analysis. Data are shown as the mean ± standard error of the mean (SEM), along with individual data points.

## Results

### Social subordination induced mouse allogrooming

Many studies focusing on social hierarchy have shown that mice at lower social status exhibit reduced aggression levels [16]. Consequently, we established social hierarchies by pair-housing males for 1 week and determining their social status by introducing intruders (Fig. S1A, Hierarchy Establish and Identification). Mice exhibiting aggression were classified as dominant, while those that did not display aggression were categorized as subordinate. Dominant mice also had a higher win rate in the tube test (Fig. S1B), indicating a degree of consistency between the social ranks identified by the intruder assay and the tube test (Fig. S1C).

**Fig. 1:**
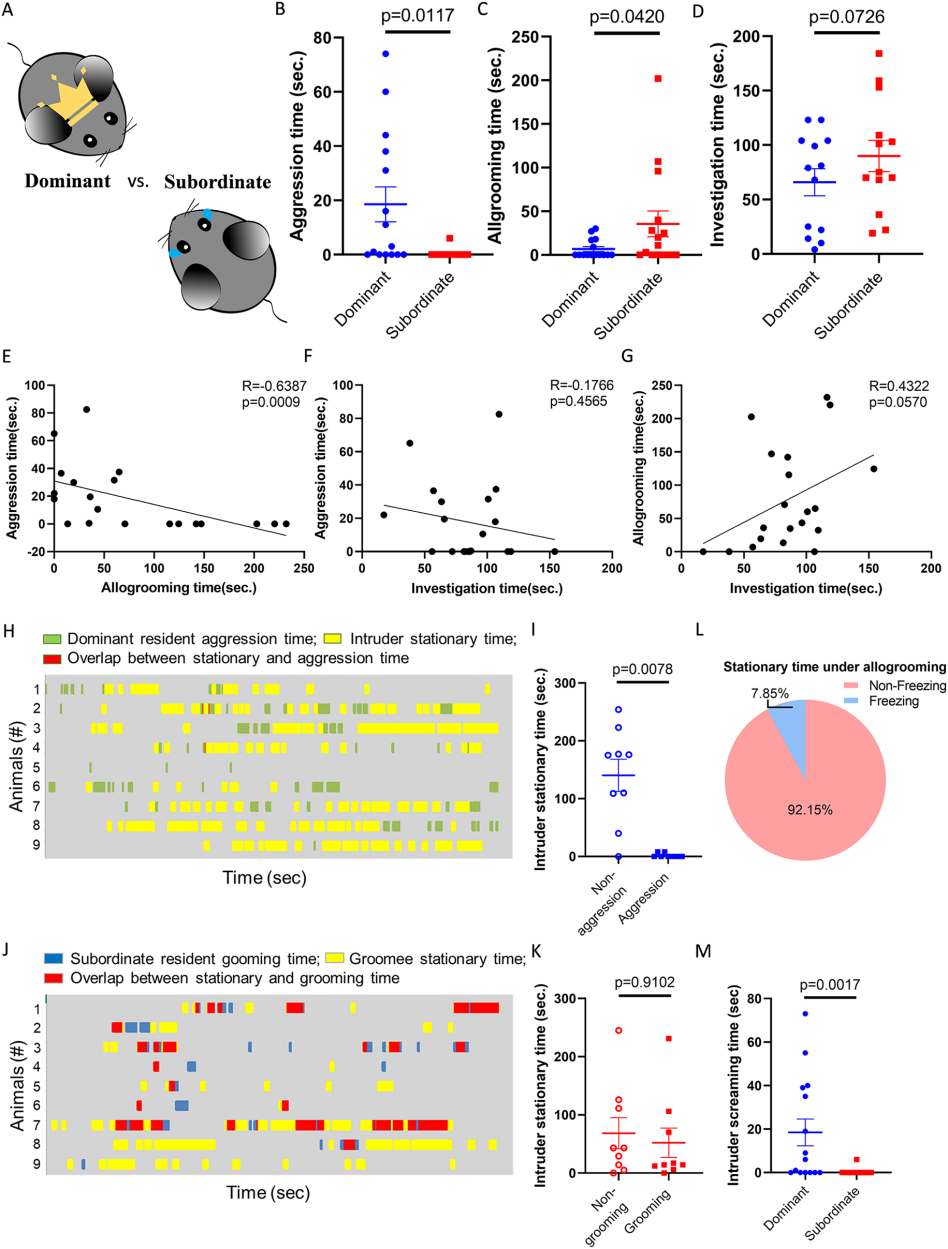
Social subordination induced mouse allogrooming. (**A**) Comparison between dominant and subordinate residents individually in the resident-intruder assay. (**B**) Aggression time of dominant or subordinate residents (Wilcoxon test, *n* = 15 pairs). (**C**) Allogrooming time of dominant or subordinate residents (Wilcoxon test, *n* = 15 pairs). (**D**) Social investigation time of dominant or subordinate residents (Paired t-test, *n* = 13 pairs). (**E**) Correlation between aggression and allogrooming time of pair-housing mice toward intruders (Spearman correlation, *n* = 20). (**F**) Correlation between aggression and investigation time of pair-housing mice toward intruders (Spearman correlation, *n* = 20). (**G**) Correlation between allogrooming and investigation time of pair-housing mice toward intruders (Spearman correlation, *n* = 20). (**H**) Raster plot presenting the aggression of dominant residents and the stationary periods of intruders (*n* = 9). (**I**) Intruders’ stationary time under aggression or nonaggression behaviors of dominant residents (Wilcoxon test, *n* = 9 pairs). (**J**) Raster plot presenting the allogrooming of subordinate residents and the stationary periods of intruders (*n* = 9). (**K**) Intruders’ stationary time under grooming or nongrooming behaviors of subordinate residents (Wilcoxon test, *n* = 9). (**L**) Percentage of intruders’ freezing-like and non-freezing-like stationary time under subordinate residents’ allogrooming (*n* = 9). (**M**) Screaming time of intruders interacting with dominant or subordinate residents (Mann‒Whitney test, *n* = 15 pairs). Mean ± SEM

After distinguishing dominant and subordinate mice, we examined their social behaviors to the intruders in the resident-intruder assay individually (Fig. 1A and S1A, Behavioral testing). As expected, dominant residents exhibited significantly higher levels of aggression toward intruders than subordinate residents (Fig. 1B and Video S1), including a longer total time and more bouts along with a shorter latency to aggression (Fig. S1D). In contrast, subordinate residents surprisingly showed significantly higher levels of allogrooming toward intruders than dominant residents, including a longer total time and more bouts (Fig. 1C, S1E and Video S2). There was no significant difference in general social interaction (Fig. 1D, S1F and Video S3). The relationships among aggression, allogrooming and general investigation in these residents were also examined. We observed a negative correlation between aggression and allogrooming (Fig. 1E), while there was no significant correlation between aggression and social investigation (Fig. 1F). Allogrooming is also positively correlated with investigation (Fig. 1G), with statistics close to significance (*p* = 0.057).

### Intruder mice responded differently to residents’ aggression and allogrooming

During the resident-intruder assay, allogrooming can sometimes be mistaken for aggression, such as biting, barbering [44], and aggressive allogrooming [45, 46], which motivation remains unclear. It was therefore crucial to distinguish the biological functions between aggression and allogrooming observed in our study. We compared the behaviors of intruders during the interactions with either dominant or subordinate residents. When intruders were subjected to aggression from dominant residents (Fig. 1H and I), they often attempted to flee and exhibited minimal stationary time during the interaction. In contrast, during grooming sessions with subordinate residents (Fig. 1J and K), intruders displayed significantly more stationary time. Additionally, only a small proportion of this stationary immobility showed stressful, freezing-like characteristics (Fig. 1L). More importantly, the screaming response, which is elicited from intruders being attacked by residents [47], was rarely detected during allogrooming by subordinate residents (Fig. 1M). These results indicated that intruders displayed distinct responses to allogrooming and aggression. Whereas aggression predominantly led to screaming and avoidance in the intruders, allogrooming elicited a calming response, as indicated by increased periods of stationary behavior in the intruders.

### Ablation of olfaction led to mouse allogrooming

**Fig. 2:**
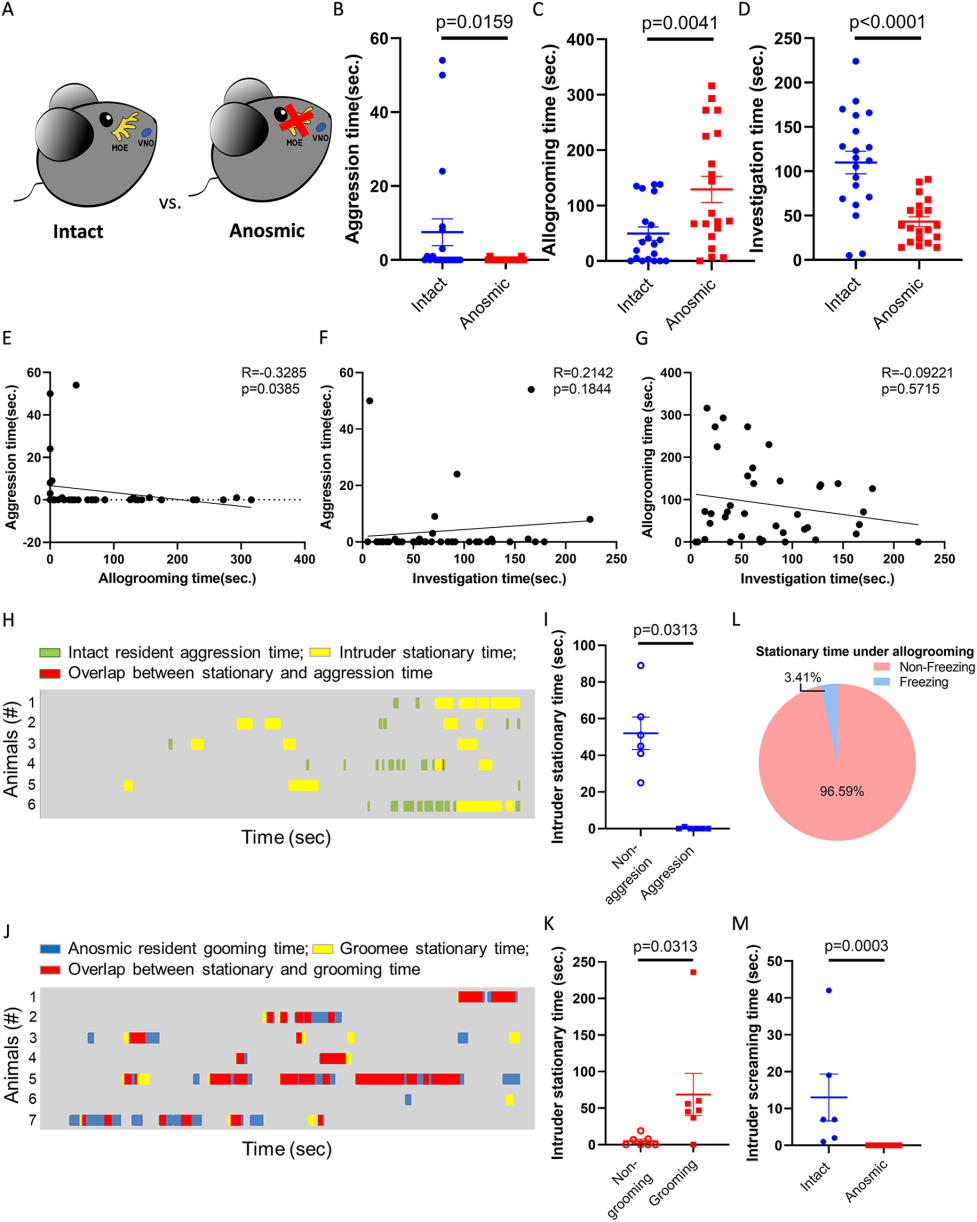
Elimination of olfaction resulted in mouse allogrooming. (**A**) Comparison between intact and anosmic residents who were treated with dichlobenil to ablate MOE in the resident-intruder assay. (**B**) Aggression time of intact or anosmic residents (Mann‒Whitney test, *n* = 20,20). (**C**) Allogrooming time of intact or anosmic residents (Mann‒Whitney test, *n* = 20,20). (**D**) Social investigation time of intact or anosmic residents (Unpaired t-test, *n* = 20,20). (**E**) Correlation between aggression and allogrooming time of intact and anosmic mice toward intruders (Spearman correlation, *n* = 40). (**F**) Correlation between aggression and investigation time of intact and anosmic mice toward intruders (Spearman correlation, *n* = 40). (**G**) Correlation between allogrooming and investigation time of intact and anosmic mice toward intruders (Spearman correlation, *n* = 40). (**H**) Raster plot presenting the aggression of intact residents and the stationary periods of intruders (*n* = 6). (**I**) Intruders’ stationary time under aggression or nonaggression behaviors of intact residents (Wilcoxon test, *n* = 6). (**J**) Raster plot presenting the allogrooming of anosmic residents and the stationary periods of intruders (*n* = 7). (**K**) Intruders’ stationary time under grooming or nongrooming behaviors of anosmic residents (Wilcoxon test, *n* = 7). (**L**) Percentage of intruders’ freezing-like and non-freezing-like stationary time under anosmic residents’ allogrooming (*n* = 7). (**M**) Screaming time of intruders interacting with intact or anosmic residents (Mann‒Whitney test, *n* = 6, 7). Mean ± SEM

In addition to social subordination, blocking pheromone-sensing is also known to eliminate mouse aggression [48, 49]. To further test whether suppressing aggressive motivation can induce allogrooming behavior, we individually housed mice and compared social behaviors toward intruders between intact and anosmic residents (Fig. 2A). Consistent with previous studies [31, 48], resident mice injected with dichlobenil for ablation of the MOE exhibited almost no aggression (Fig. 2B and S3A). More importantly, dichlobenil treatment led to a significant increase in allogrooming and a decrease in social investigation (Fig. 2C-D and S3B-C). Consequently, aggression and allogrooming showed a negative correlation (Fig. 2E), whereas no significant correlation was observed with social investigation (Fig. 2F and G). These results suggest that, in addition to social subordination, the loss of aggression through olfactory system disruption can also induce allogrooming.

For intruders’ responses, similar to the comparison between intruders interacting with dominant or subordinate residents, intruders spent almost no stationary time during aggressive encounters with intact residents (Fig. 2H-I). In contrast, they remained mostly still while being groomed by anosmic residents (Fig. 2J-K), with only a small proportion of this stationary time showing freezing-like characteristics (Fig. 2L).No screaming was detected during allogrooming by anosmic residents (Fig. 2M).

### Allogrooming served as a prosocial behavior that provides cleaning and consolation

**Fig. 3:**
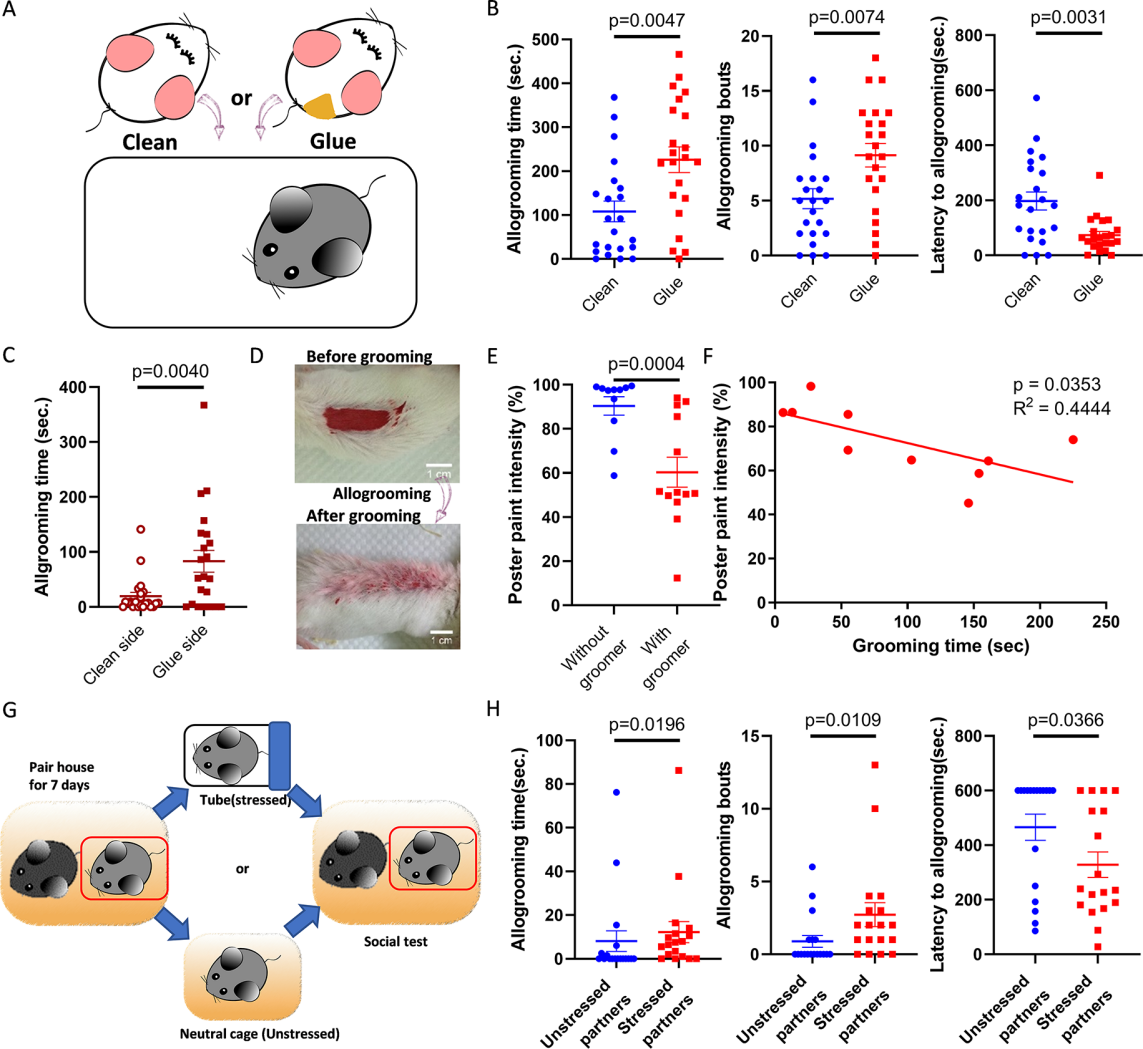
Groomers engaged in allogrooming to clean and comfort the recipients. (**A**) Comparison of responses of residents treated with dichlobenil to clean intruders or intruders covered with unfamiliar materials (glue). (**B**) Allogrooming time, bouts and latency of anosmic residents to intruders with or without stick glue (time and latency, Mann‒Whitney test; bouts, Unpaired t-test, *n* = 22 pairs). (**C**) Allogrooming time on clean or glued sites of intruders (Wilcoxon test, *n* = 22 pairs). (**D**) Representative images showing the poster paint on anesthetized intruders before or after allogrooming provided by residents. (**E**) The paint intensity with or without groomer interaction (Mann‒Whitney test, *n* = 11,13). (**F**) Correlation between paint intensity and allogrooming time (Pearson correlation, *n* = 10). (**G**) Experimental scheme testing residents’ responses to distressed social partners. (**H**) The allogrooming time, bouts and latency of residents interacting with unstressed or distressed partners (Mann‒Whitney test, *n* = 18 pairs). Mean ± SEM

Allogrooming has been widely recognized as a prosocial behavior because groomers clean and comfort the recipients [2]; however, the underlying motivation in our mouse study requires further validation. Our investigation first focused on the responses of residents treated with dichlobenil to intruders covered with unfamiliar tactile textures, such as glue from a glue stick (Fig. 3A). The addition of glue significantly increased allogrooming time and bouts among anosmic residents, with a shorter latency to allogrooming (Fig. 3B). In addition, residents exhibited a preference for grooming the glued side of intruders (Fig. 3C). Similar results can also be observed in intruders covered with mineral oil (Fig. S4A-C). Neither of glue and oil was preferred in a two-choice assay (Fig. S4D-F), suggesting that the increase in allogrooming is not due to their preference for these materials. Furthermore, we applied poster paint to anesthetized mice (to prevent self-grooming) and assessed the intensity of the remaining paint after these mice were introduced as intruders in the presence or absence of resident mice. Not surprisingly, allogrooming performed by residents significantly reduced the presence of paint on intruders (Fig. 3D and E). We also identified a negative correlation between paint intensity and grooming time (Fig. 3F), supporting the notion that allogrooming contributes to the hygienic maintenance of recipients.

In addition to hygiene, previous studies have shown that mice engage in allogrooming to comfort their distressed social partners [26, 27]. Consistent with these findings, our study revealed that mice showed increased allogrooming toward distressed partners subjected to 30 min of restraint stress (Fig. 3G-H), implying a motivation to comfort social partners. Taken together, by comprehensively examining the stationary behaviors of intruders, assessing the cleaning effect, and evaluating the response of residents toward distressed partners, we believed that the allogrooming observed in this study provided benefits to the intruders and functioned as a prosocial interaction.

### Suppressing aggression circuits enhanced mouse allogrooming

**Fig. 4:**
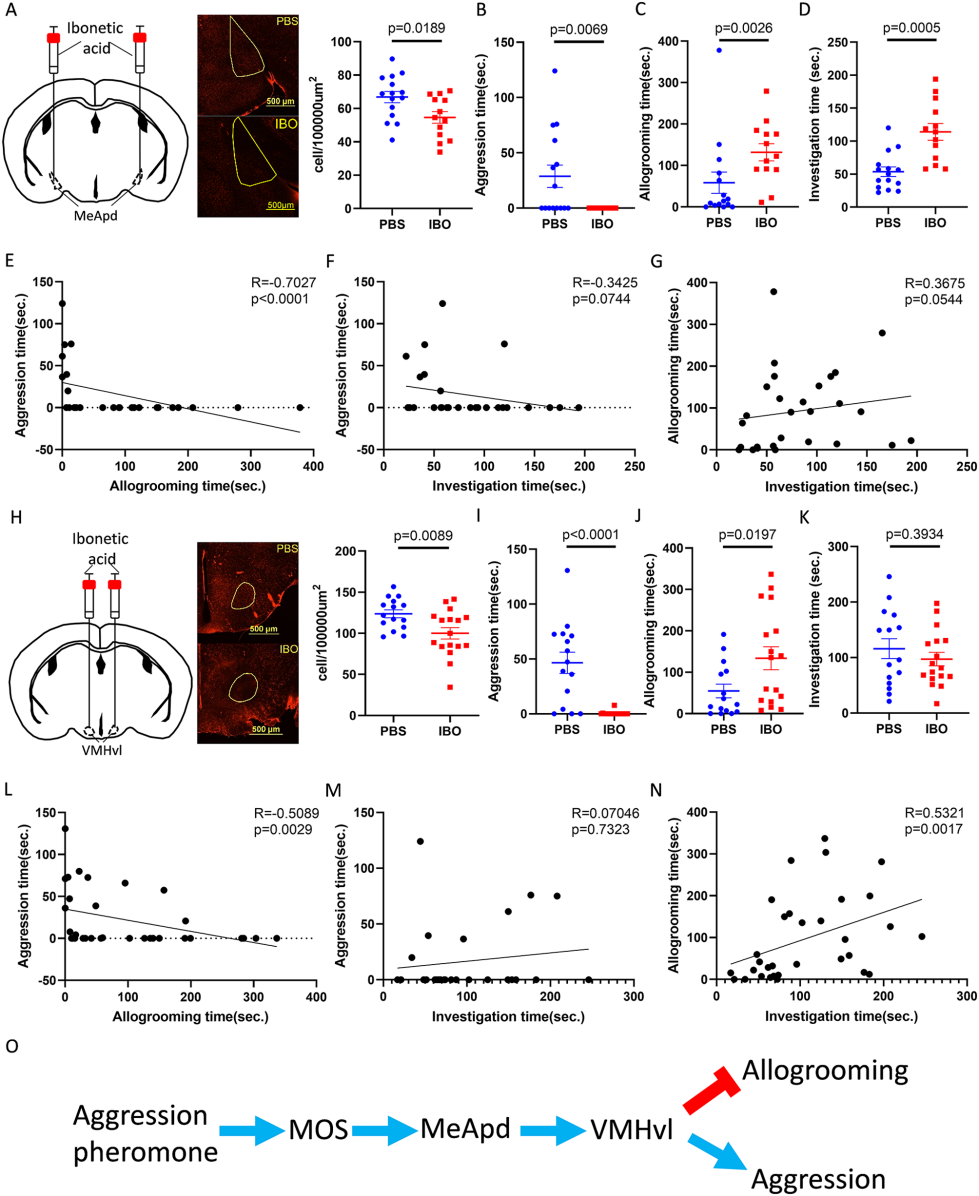
Suppressing aggression circuits enhanced mouse allogrooming. (**A**) Representative images and the density of neurons in MeApd injected with ibotenic acid (IBO) for lesion or PBS as control (Unpaired t-test, *n* = 15,13). (**B**) Aggression time of residents with PBS or IBO injection in MeApd (Mann‒Whitney test, *n* = 15,13). (**C**) Allogrooming time of residents with PBS or IBO injection in MeApd (Unpaired t-test, *n* = 15,13). (**D**) Social investigation time of residents with PBS or IBO injection in MeApd (Unpaired t-test, *n* = 15,13). (**E**) Correlation between aggression and allogrooming time of MeApd sham and lesion mice toward intruders (Spearman correlation, *n* = 28). (**F**) Correlation between aggression and investigation time of MeApd sham and lesion mice toward intruders (Spearman correlation, *n* = 28). (**G**) Correlation between allogrooming and investigation time of MeApd sham and lesion mice toward intruders (Spearman correlation, *n* = 28). (**H**) Representative images and the density of neurons in VMHvl injected with ibotenic acid (IBO) for lesion or PBS as control (Mann‒Whitney test, *n* = 15,17). (**I**) Aggression time of residents with PBS or IBO injection in the VMHvl (Mann‒Whitney test, *n* = 15,17). (**J**) Allogrooming time of residents with PBS or IBO injection in the VMHvl (Mann‒Whitney tes, *n* = 15,17). (**K**) Social investigation time of residents with PBS or IBO injection in the VMHvl (Unpaired t-test, *n* = 15,17). (**L**) Correlation between aggression and allogrooming time of VMHvl sham and lesion mice toward intruders (Spearman correlation, *n* = 32). (**M**) Correlation between aggression and investigation time of VMHvl sham and lesion mice toward intruders (Spearman correlation, *n* = 32). (**N**) Correlation between allogrooming and investigation time of VMHvl sham and lesion mice toward intruders (Spearman correlation, *n* = 32). (**O**) Inhibitory role of aggression circuits in mouse allogrooming. Mean ± SEM

Both subordination and olfactory ablation decreased aggression and induced allogrooming. To further investigate how aggression inhibits allogrooming, we used ibotenic acid (IBO) to chemically ablate MeApd (Fig. 4A), which is known to be involved in both aggression and allogrooming [26, 50]. After two weeks of recovery from the chemical lesion under single housing, mice showed almost no aggression during intruder assay (Fig. 4B and S5A). Allogrooming and social investigation, however, were both increased compared to the control mice that received PBS injection (Fig. 4C-D and S5B-C). A negative correlation was observed between aggression and allogrooming but not in other relationships (Fig. 4E-G).

Subsequently, we examined mice with lesions in the VMHvl (Fig. 4H), which receives projections from MeApd and regulates aggression [51]. Similar to the manipulation in MeApd, ablation of the VMHvl reduced aggression but increased allogrooming (Fig. 4I-J and S5D-E), while there was no significant change in social investigation (Fig. 4K and S5F). Once again, aggression and allogrooming showed a negative correlation (Fig. 4L). Social investigation also positively correlated with allogrooming but not aggression (Fig. 4M-N).

To summarize, by examining the social behaviors of non-aggressive mice (Table S1), our results revealed a negative relationship between aggression and allogrooming. Suppressing aggression through subordination, olfactory ablation, and lesions of aggressive neural substrates all led to increased voluntary allogrooming. These findings suggest that aggressive circuits, from the olfactory system to the MeApd and VMHvl, play an inhibitory role in regulating allogrooming behavior (Fig. 4O).

### Negative relationships between aggression and prosocial behaviors in preschool children

**Fig. 5:**
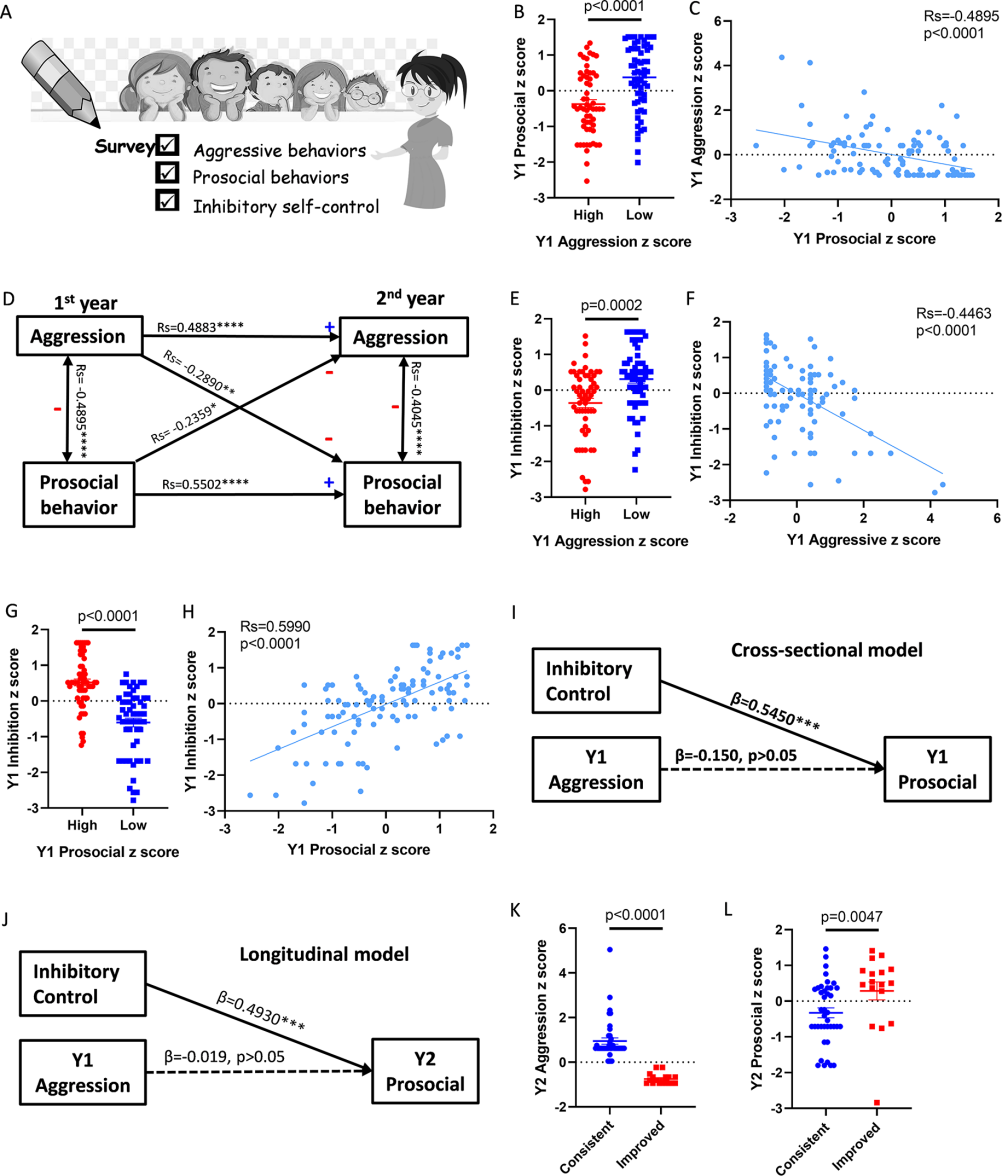
Inhibitory control ability modulates negative relationship between aggression and prosocial behaviors in preschool children. (**A**) Teachers’ evaluation of children’s behaviors in the 1^st^ (Y1) and the 2^nd^ (Y2) years. (**B**) Prosocial z score of children with positive (high) or negative (low) aggression z score (Mann‒Whitney, *n* = 57,61). (**C**) Correlation between prosocial and aggression z scores (Spearman correlation, *n* = 118). (**D**) The cross-sectional and longitudinal relationships between aggression and prosocial behaviors. (**E**) Self-inhibition z score of children with positive (high) or negative (low) aggression z score (Mann‒Whitney, *n* = 57,61). (**F**) Correlation between self-inhibition and aggression z score (Spearman correlation, *n* = 118). (**G**) Self-inhibition z score of children with positive (high) or negative (low) prosocial z scores (Mann‒Whitney, *n* = 62,56). (**H**) Correlation between self-inhibition and prosocial z score (Spearman correlation, *n* = 118). (**I**) Cross-sectional model for the effect of aggression on prosocial behaviors after controlling inhibitory self-control (Hierarchical regression analysis, *n* = 118). (**J**) Longitudinal model for the effect of aggression on prosocial behaviors after controlling inhibitory self-control (hierarchical regression analysis, *n* = 118). (**K**) Y2 aggression z score in the consistent group (positive aggression score in both years) or improved group (positive aggression score in Y1 but negative aggression score in Y2) (Mann‒Whitney test, *n* = 40,17). (**L**) Y2 prosocial z score in the consistent group or improved group (Mann‒Whitney test, *n* = 40,17). Mean ± SEM

The findings in mice inspired us to investigate the relationship between aggression and prosocial behaviors across species, especially seeking for possible application value in humans. We recruited 118 4-year-olds preschoolers who were new to school and used the Aggressive Behavior Subscale of the Preschool Social Behavior Scale and the Preschool Prosocial Behavior Scale to examine their aggressive (overt and relational aggression) and prosocial (sharing, helping, caring and cooperation) behaviors (Fig. 5A) [40, 42]. Similar to our mouse findings, young children with high aggressiveness (z score > 0) showed significantly lower prosocial behaviors (Fig. 5B). We also identified a strong negative correlation between aggression and prosocial behaviors (Fig. 5C). Specifically, both forms of aggressive behaviors were negatively correlated with all forms of prosocial behaviors (Table S2).

As it’s challenging to infer causal relationships from correlational associations, we conducted a cross-lagged research design to further examine the potential mutual relationship by observing these children’s behaviors one year apart. The cross-sectional analysis at both time points of the cross-lagged design helped us examine the robustness of the correlational pattern of these behaviors. The relationship between aggressive and prosocial behavior in the second year (Y2) was identical to that in the first year (Y1). Aggressive and prosocial behaviors remained negatively correlated after one year (Fig. S6A and B). Next, the longitudinal analysis of the cross-lagged design further allowed us to explore the possible directional influence with temporal logic. Y1 prosocial behaviors were positively correlated with Y2 prosocial behaviors and negatively correlated with Y2 aggression (Table S3). Similarly, Y1 aggression was positively correlated with Y2 aggression and negatively correlated with Y2 prosocial behaviors (Table S3), indicating that both aggression and prosocial behaviors are stable over time and maintain negative correlations with each other in the long term (Fig. 5D).

### Inhibitory control ability modulates children’s aggression and prosocial behaviors

Although we cannot invasively inhibit children’s aggressive behaviors, as we have done in mice, the assessment of executive functions allowed us to evaluate children’s inhibitory self-control ability and its relationship with aggressive or prosocial behaviors [43]. The data suggested that, in both the first and the second years, children with high aggressiveness showed worse inhibitory self-control (Fig. 5E, F and S6C, D), while children with high prosocial behavior showed better inhibitory self-control (Fig. 5G, H and S6E, F). The findings implied that inhibitory self-control may help children inhibit their aggressive motivation and thus more easily perform prosocial behaviors.

Next, we performed hierarchical regression analysis to statistically control the effect of inhibitory self-control to investigate how aggression modulates prosocial behaviors through inhibitory self-control (Table S4). After excluding the effect of inhibition, the cross-sectional correlation between aggression and prosocial behavior in the first year was no longer significant (Fig. 5I). For the longitudinal relationship, controlling inhibitory self-control also eliminated the significant correlation between Y1 aggression and Y2 prosocial behavior (Fig. 5J), suggesting the pivotal role of inhibitory self-control in the effect of aggression on prosocial behavior.

In addition to examining the influence of internal inhibition, we found some children improved their behaviors from high aggression to low aggression over the year (z score from positive to negative). Since controlling aggressive behavior is a crucial focus of school education at this age [52], behavioral change over this year gave us an opportunity to further examine the consequences of inhibiting aggression through external intervention. In contrast to the children maintaining their high aggressiveness (consistent group), these children with behavioral switching (improved group) after one year of school education showed not only decreased aggression but also increased prosocial behaviors in the second year (Fig. 5K and L), once again supporting the negative association between aggressive and prosocial behaviors and implying the possibility of inhibiting aggression through education. Together, by examining the behaviors of preschool children through a longitudinal survey study, we found negative associations between aggressive and prosocial behaviors in both short- and long-term relationships. This association appears to hinge on self-inhibitory control ability and the inhibition of external intervention.

## Discussion

Due to the limited research on allogrooming, the relationship between aggression and allogrooming has not been previously investigated systematically in house mice. Using the resident-intruder assay to examine both behaviors simultaneously, our studies revealed a negative correlation between aggression and allogrooming. Additionally, inhibiting aggressive motivation—achieved through social subordination, olfaction ablation, or the suppression of aggressive circuits—resulted in voluntary allogrooming behavior by resident mice towards intruders. This suggests an inhibitory influence of aggression circuits on allogrooming during the resident-intruder assay. It is important to note that the negative relationship between aggression and prosocial allogrooming should not be assumed. The relationship between these two behaviors could be positive or show no correlation. Furthermore, manipulations inhibiting aggression could also inhibit or have no influence on allogrooming. Although our finding may seem intuitive to some, this assumption should not be taken for granted until it is experimentally demonstrated.

During social interactions, mice may sometimes aggressively perform allogrooming, and some studies have classified this behavior as aggressive [45, 46]. This is why, despite the long recognition of allogrooming as a prosocial behavior in many rodent studies, our research has placed significant emphasis on distinguishing the observed allogrooming from aggression. We first demonstrated that recipients responded distinctly to aggression versus allogrooming: recipients typically sought to avoid or escape from aggression, often accompanied by screaming, while during allogrooming, they mostly remained still. Notably, while intruders’ freezing behavior can sometimes be observed after, but not during, aggressive interactions, the immobility seen with allogrooming occurred during the interaction and exhibited minimal freezing-like characteristics. Additionally, we presented evidence for the prosocial motivations behind allogrooming, such as cleaning and comforting social partners. Together, these findings are intended to reduce the likelihood of aggressive motivations in the allogrooming we observed. Nevertheless, distinguishing between aggressive and normal allogrooming remains challenging, and a clearer definition in future research could help to further clarify the underlying motivations behind aggressive allogrooming.

There have been a few studies using other rodent species, like voles, rats, and spiny mice, to address ethological questions about allogrooming [20–25]. For example, it has been shown that lesioning the bilateral amygdala in male Mongolian gerbils reduces aggressive behavior and increases allogrooming [53]. However, in contrast to the negative interaction of aggression, allogrooming with positive valence has been understudied from a mechanistic perspective in house mice. Interestingly, a recent study focusing on mouse consolation behavior showed increased allogrooming after MeA stimulation [26], contrasting our findings regarding increased allogrooming in MeApd-ablated mice. These divergent results may be attributed to targeting different subdivisions or neural populations within the MeA. Nonetheless, our study concluded that MeApd plays an inhibitory role in allogrooming through aggressive motivation without excluding the possibility of other modulatory functions in allogrooming or consolation behavior. Furthermore, our study demonstrated that allogrooming can be easily induced in mice using various approaches, providing simple platforms for future research on the interplay between aggression and allogrooming in the MeA and other brain regions.

Inspired by the mouse findings, we conducted a longitudinal follow-up study of more than a hundred young children and yielded similar conclusions as the mouse study. Both cross-sectional and longitudinal data suggested negative correlations between aggressive and prosocial behaviors of preschool children, who have minimal school education and social experience with peers. More importantly, this developmental stage, typically between 3 and 4 years old, marks the gradual differentiation of human inhibitory control as a distinct component from the earlier mixed self-regulation abilities [54]. While previous studies have indicated the importance of inhibitory control in controlling aggression [55], our results highlighted its potential role in facilitating the performance of prosocial behaviors. In other words, intrinsic and extrinsic mechanisms that inhibit aggression may create opportunities for the emergence and development of prosocial behaviors, particularly during the crucial early stages of social development.

Previous research has shown that both aggression and prosocial behaviors are the main strategies preschool children use to resolve social conflicts [56]. Although children instinctively generate aggression and prosocial behaviors early in life [57, 58], demonstrating appropriate prosocial behaviors in a group requires more cognitive resources and further learning [59–61]. In the absence of external pressure, children often default to exploit less cognitively demanding strategies, like aggression [62]. However, when external environments impose rules or expectations to curb aggression—such as the prohibition of violence in kindergartens—children, even those with poorer self-control, are more likely to adopt prosocial strategies, thereby fostering the development of these behaviors. Thus, while biological development has an impact over this year, school education may serve as a powerful external factor that suppresses aggression and encourages the adoption of prosocial behaviors among young children. Consequently, this study enlightens us that helping children develop self-control in the early years and receive appropriate early childhood education can effectively reduce aggression and improve prosocial behaviors, which is critical for the early and long-term development of social competence.

The present studies, based on mouse and human data, suggested the inhibitory role of aggression motivation on prosocial behaviors. Although our results provided valuable insight into human and animal social interactions, there are still certain limitations to our findings. For example, despite the correlation and hierarchical regression analyses implying the importance of inhibitory control in children’s prosocial behaviors, the causality between these two characteristics remains to be investigated. Even though our mouse model demonstrated that silencing aggression can induce allogrooming, drawing the same conclusion in humans still requires cautious consideration. Second, although we have provided multiple evidence from both the groomers’ and recipients’ perspectives to support the prosocial nature of the observed behaviors, entirely excluding the possibility of aggressive components in allogrooming may require the development of an automated imaging analysis system for more comprehensive behavioral analysis in future research. Lastly, our mouse experiments ablated neurons of multiple brain regions without targeting specific neuron populations. Given that the compositions of neurons in most brain regions are heterogeneous, understanding the specific function of these targeted regions in allogrooming requires further study. Our understanding of allogrooming in mice remains limited. Questions about which neural substrates regulate this behavior, how aggression circuits inhibit it, and how environments and hormones regulate it, still persist. We believe that the simple platform established in this study opens a new avenue for investigating these questions, aiding us in exploring mouse prosocial motivations and understanding the relationship between aggression and prosocial behaviors in future research.

## Conclusions

In summary, our study revealed the negative relationship between aggression and allogrooming and demonstrated the inhibitory function of aggression circuits in mouse allogrooming. We also established simple platforms of allogrooming for future research on mouse prosocial behavior. More importantly, mouse results led us to reveal negative relationships between aggression and prosocial behaviors in preschool children and a potential role of inhibitory self-control and school education in modulating both types of behaviors. While mouse studies allowed us to investigate mechanistic questions that are not feasible in human subjects, extended exploration in children further underscore the biological significance of the discoveries. The combination of findings from these two species therefore offers complementary perspectives on the phenomenon, providing new insights into animal and human social interactions with potential implications for parenting and preschool education.

## Electronic supplementary material

Below is the link to the electronic supplementary material.

Supplementary Material 1

Supplementary Material 2

Supplementary Material 3

Supplementary Material 4

## Data Availability

No datasets were generated or analysed during the current study.
