# Supplementary material for: Uncovering hidden prosocial behaviors underlying aggression motivation in mice and young children
Source: Behav Brain Funct. 2024 Nov 28;20:32. doi: 10.1186/s12993-024-00260-z (PMC11605964; doi:10.1186/s12993-024-00260-z)
Supplement: Supplementary file 4 — Supplementary Material 4 [file 12993_2024_260_MOESM4_ESM.docx]

**Supplementary Figures**

**Supplementary Figure 1. Dominant and subordinate mice established by social hierarchy were tested individually in the resident-intruder assay.** (A) Eight-week-old mice were housed in pairs for 1 week to establish hierarchy. In order to identify social rank, one intruder was introduced into the cage with paired residents for a 10-min interaction. The resident displaying aggression was identified as a dominant male; the resident showing no aggression was identified as a subordinate male. The identified dominant and subordinate males were tested individually in the standard resident-intruder assay to study social interaction. (B) Tube test winning rate of dominant and subordinate mice defined by resident-intruder assay (Mann‒Whitney test, n=14,14). (C) Contingency table for the relationship between social ranks defined by the resident-intruder assay and the tube test (Fisher’s exact test, n=28). (D) Aggression bouts and latency to aggression of dominant or subordinate residents (Wilcoxon test, n=15 pairs). (E) Allogrooming bouts and latency to allogrooming of dominant or subordinate residents (Wilcoxon test, n=15 pairs). (F) Social investigation bouts and latency to investigation of dominant or subordinate residents (paired t-test, n=13).

**Supplementary Figure 2. Stereotaxic surgery significantly decreased aggression and increased allogrooming in C57BL/6J but not in C57BL/6N mice.** (A) Comparison of C57BL/6J males with or without stereotaxic surgery (PBS injection into MeApd) (B) Aggression time of intact or PBS-injected C57BL/6J residents (Mann‒Whitney test, n=20,17). (C) Allogrooming time of intact or PBS-injected C57BL/6J residents (Mann‒Whitney test, n=20,17). (D) Social investigation time of intact or PBS-injected C57BL/6J residents (Mann‒Whitney test, n=20,17). (E) Comparison of C57BL/6N males with or without stereotaxic surgery (PBS injection into MeApd) (F) Aggression time of intact or PBS-injected C57BL/6N residents (Mann‒Whitney test, n=10,15). (G) Allogrooming time of intact or PBS-injected C57BL/6N residents (Mann‒Whitney test, n=10,15). (H) Social investigation time of intact or PBS-injected C57BL/6N residents (Mann‒Whitney test, n=10,15).

**Supplementary Figure 3. Bouts and latency of aggression, allogrooming, and social investigation in intact and anosmics mice.** (A) Aggression bouts and latency to aggression of intact or anosmic residents (Mann-Whitney test, n=20,20). (B) Allogrooming bouts and latency to allogrooming of intact or anosmic residents (Mann-Whitney test, n=20,20). (C) Social investigation bouts and latency to investigation of intact or anosmic residents (bouts: unpaired test, n=20,20; latency: Mann-Whitney test, n=20,20).

**Supplementary Figure 4. Mineral oil on intruders induced residents’ allogrooming.** (A) Comparison of anosmic residents’ responses to clean intruders or intruders covered with unfamiliar materials (mineral oil). (B) Allogrooming time, bouts and latency of residents to intruders with or without mineral oil (Mann‒Whitney test, n=16,16). (C) Allogrooming time on clean and oiled sites of intruders (Wilcoxon test, n=16). (D) Two-choice assay: a mouse was introduced into a cage to explore a stimulus (glue from a glue stick or mineral oil) and PBS on two opposite walls. (E) Investigation time of mice toward PBS or glue (Wilcoxon test, n = 21). (F) Investigation time of mice toward PBS or mineral oil (Wilcoxon test, n = 21). Mean ± SEM.

**Supplementary Figure 5.** **Bouts and latency of aggression, allogrooming, and social investigation in MeApd lesion, VMHvl lesion and PBS control mice.** (A) Aggression bouts and latency of residents with PBS or IBO injection in MeApd (Mann‒Whitney test, n=15,13). (B) Allogrooming bouts and latency of residents with PBS or IBO injection in MeApd (Mann‒Whitney test, n=15,13). (C) Social investigation bouts and latency of residents with PBS or IBO injection in MeApd (Mann‒Whitney test, n=15,13). (D) Aggression bouts and latency of residents with PBS or IBO injection in VMHvl (Mann‒Whitney test, n=15,17). (E) Allogrooming bouts and latency of residents with PBS or IBO injection in VMHvl (Mann‒Whitney test, n=15,17). (F) Social investigation bouts and latency of residents with PBS or IBO injection in VMHvl (Mann‒Whitney test, n=15,17).

**Supplementary Figure 6. Self-inhibitory control was negatively correlated with aggression but positively correlated with prosocial behaviors of preschool children in the 2^nd^ year.** (A) Prosocial z score of children with positive (high) or negative (low) aggression z score in the 2^nd^ year (Mann‒Whitney, n=57,61). (B) Correlation between prosocial and aggression z score in children in the 2^nd^ year (Spearman correlation, n=118). (C) Self-inhibition z score of children with positive (high) or negative (low) aggression z score in the 2^nd^ year (Mann‒Whitney, n=57,61). (D) Correlation between Self-inhibition and aggression z score in children in the 2^nd^ year (Spearman correlation, n=118). (E) Self-inhibition z score of children with positive (high) or negative (low) prosocial z score in the 2^nd^ year (Mann‒Whitney, n=62,56). (F) Correlation between Self-inhibition and prosocial z score in children in the 2^nd^ year (Spearman correlation, n=118). Mean ± SEM.

**Table S1. Summary of Figures, Strains, Manipulations, Results, and Sample Sizes in Mouse Experiments**

| **Figures** | **Residents** | | **Intruders** | | **Residents’ responses** | | | **Sample size (n)** |
| --- | --- | --- | --- | --- | --- | --- | --- | --- |
|  | **Strain** | **Manipulation** | **Strains** | **Status** | **Aggression** | **Allogrooming** | **Investigation** | **Experiment/Control** |
| 1A-1D,  S1D-S1F | C57BL/6J | **Subordination** | BALB/c | Olfactory Ablation | ↓ | ↑ | - | 15/15 (1B-C, S1D-E)  13/13 (1D, S1F) |
| 2A-2D, S3A-S3C | C57BL/6J | **Olfactory Ablation** | BALB/c | Olfactory Ablation | ↓ | ↑ | ↓ | 20/20 |
| 4A-4D, S5A-S5C | C57BL/6N | **MeApd Lesion** | BALB/c | Olfactory Ablation | ↓ | ↑ | - | 13/15 |
| 4H-4K, S5D-S5F | C57BL/6N | **VMHvl Lesion** | BALB/c | Olfactory Ablation | ↓ | ↑ | - | 17/15 |
| S2A-S2D | C57BL/6J | **Stereotax sham surgery** | BALB/c | Olfactory Ablation | ↓ | ↑ | ↑ | 17/20 |
| S2E-S2H | C57BL/6N | **Stereotax sham surgery** | BALB/c | Olfactory Ablation | - | - | - | 15/10 |
| **Figures** | **Strain** | **Status** | **Strains** | **Manipulation** | **Aggression** | **Allogrooming** | **Investigation** | **Experiment/Control** |
| 3A-3C | C57BL/6J | Olfactory Ablation | BALB/c | Olfactory Ablation,  **Glue-painted,** Anesthetized | - | ↑ | - | 22/22 |
| S4A-S4C | C57BL/6J | Olfactory Ablation | BALB/c | Olfactory Ablation,  **Oil-painted,** Anesthetized | - | ↑ | - | 16/16 |
| 3G-3H | C57BL/6J | Pair-housed | C57BL/6J | Pair-housed, **Restrained** | - | ↑ | - | 18/18 |
| **Figure** | **Strain** | **Manipulation** | **Strain** | **Status** | **Intruder’s response** | | | **Experiment** |
| 1H-1I, 1M | C57BL/6J | **Dominant** | BALB/c | Olfactory Ablation | Rarely stayed still and frequently screamed under attack. | | | 9 |
| 1J-1L, 1M | C57BL/6J | **Subordinate** | BALB/c | Olfactory Ablation | Preferred to remain still and rarely screamed when groomed. | | | 9 |
| 2H-2I, 2M | C57BL/6J | **Intact** | BALB/c | Olfactory Ablation | Rarely stayed still and frequently screamed under attack. | | | 6 |
| 2J-2L, 2M | C57BL/6J | **Olfactory Ablation** | BALB/c | Olfactory Ablation | Preferred to remain still and rarely screamed when groomed. | | | 7 |
| **Figures** | **Strain** | **Status** | **Strains** | **Manipulation** | **Intruder’s response** | | | **Experiment/Control** |
| 3D-3E | C57BL/6J | Olfactory Ablation | BALB/c | Olfactory Ablation, **Red paint-painted** Anesthetized, | The red paint was being cleaned by the residents. | | | 13/11 |

**Table S2. Correlations between aggression and pro-social behaviors in subscale.**

| **N=118** | **Sharing**  **z score** | **Helping**  **z score** | **Caring**  **z score** | **Cooperation**  **z score** | **Prosocial** **Behavior** **z score** |
| --- | --- | --- | --- | --- | --- |
| **Overt**  **Aggression**  **z score** | -0.4731**** | -0.4615**** | -0.4161**** | -0.4757**** | -0.4699**** |
| **Relational**  **Aggression**  **z score** | -0.3142*** | -0.3327*** | -0.2622** | -0.3232*** | -0.3179*** |
| **Total**  **Aggression**  **z score** | -0.4499**** | -0.4521**** | -0.3931**** | -0.4624**** | -0.4895**** |

Note: N=118, Spearman correlation test, p<0.01**, p<0.001***, p<0.0001****

**Table S3. Correlation between aggression and prosocial behaviors in the 1^st^ and 2^nd^ year.**

| **N=118** | **Total Aggression**  **z score 1^st^ year** | **Total Aggression**  **z score 2^nd^ year** | **Prosocial Behavior**  **z score 1^st^ year** | **Prosocial Behavior**  **z score 2^nd^ year** |
| --- | --- | --- | --- | --- |
| **Total Aggression**  **z score 1^st^ year** | ━ |  |  |  |
| **Total Aggression**  **z score 2^nd^ year** | 0.4883**** | ━ |  |  |
| **Prosocial Behavior**  **z score 1^st^ year** | -0.4895**** | -0.2359* | ━ |  |
| **Prosocial Behavior**  **z score 2^nd^ year** | -0.2890** | -0.4045**** | 0.5502**** | ━ |

Note: N=118, Spearman correlation test, P<0.05*, p<0.01**, p<0.0001****

**Table S4 Hierarchical regressions analysis of aggression on prosocial behavior (controlling for inhibition)**

|  | $\boldsymbol{\beta}$ | | | | *t* value | | $\boldsymbol{\Delta}\boldsymbol{R}^{\boldsymbol{2}}$ | *F* change |
| --- | --- | --- | --- | --- | --- | --- | --- | --- |
| Reg1(cross-sectional)：1^st^ year-aggression on 1^st^-year prosocial behavior (controlling for inhibition) | | | | | | | | |
| Step1: inhibition | | | | .622 | | 8.564*** | .387 | 73.249*** |
| Step2: inhibition | | | .545 | | | 6.484*** | .016 |  |
| aggression | | | -.150 | | | -1.780 |  | 3.169 |
| Reg2(longitudinal)： 1^st^-year aggression on 2^nd^-year prosocial behavior (controlling for inhibition) | | | | | | | | |
| Step1: inhibition | | .503 | | | 6.273*** | | .253 | 39.353*** |
| Step2: inhibition | | .493 | | | 5.245*** | | .000 |  |
| aggression | | -.019 | | | -.205 | |  | .042 |

Note: N=118, Hierarchical regression, p<0.001***
